# Neglected Intestinal Parasites, Malnutrition and Associated Key Factors: A Population Based Cross-Sectional Study among Indigenous Communities in Sarawak, Malaysia

**DOI:** 10.1371/journal.pone.0170174

**Published:** 2017-01-17

**Authors:** Yamuna Rajoo, Stephen Ambu, Yvonne Ai Lian Lim, Komalaveni Rajoo, Siew Chang Tey, Chan Woon Lu, Romano Ngui

**Affiliations:** 1 International Medical University, Bukit Jalil, Kuala Lumpur, Malaysia; 2 Department of Parasitology, Faculty of Medicine, University of Malaya, Kuala Lumpur, Malaysia; 3 Hospital Sarikei, Jalan Rentap, Sarikei, Sarawak, Malaysia; Universidade de Aveiro, PORTUGAL

## Abstract

Intestinal parasitic infections (IPIs) have been recognized as one of the most significant causes of illness among disadvantaged communities. Many studies have been conducted on the prevalence of IPIs in Malaysia. However, these studies mostly focused on the indigenous groups in Peninsular Malaysia. The present study was conducted to provide the current baseline data on prevalence of IPIs, anaemia, malnutrition and associated risk factors among the indigenous communities in Sarawak, situation at northwest Borneo island of Malaysia. A cross sectional study was conducted among the longhouses communities. Stool samples were obtained and examined for the presence of IPIs using microscopy technique. Haemoglobin measurement was done using a portable haemoglobin analyzer. Malnutrition (i.e., stunting, underweight and wasting) was assessed using the WHO Anthro software. Statistical analysis was carried out using SPSS software. A total of 341participants took part in this study. The overall prevalence of IPIs was 57.5%. Multivariate analysis indicated that the absence of toilets (OR = 1.6; 95% CI = 1.1–2.7; *p* = 0.002) and close contact with animals (OR = 1.8; 95% CI = 1.3–2.9; *p* = 0.027) as significant predictors for IPIs. The incidence of anaemia was 36.4%. The incidence of underweight, wasting and stunting were 22.2%, 5.6% and 35.4%, respectively. Multivariate analysis demonstrated that low level of parental education attainment (OR = 1.9; 95% CI = 1.2–3.0; *p* = 0.006) was identified as significant predictor for anaemia. The incidence of wasting was significantly associated with mild anaemia (OR = 1.2; 95% CI = 0.9–1.7; *p* = 0.024). Low household income was identified as significant predictor for stunting (OR = 2.1; 95% CI = 9.8–22.2; *p* = 0.001) and underweight (OR = 1.9; 95% CI = 5.6–18.7; *p* = 0.037), respectively. Essentially, the present study highlighted that intestinal parasitic infections, anaemia and malnutrition are still prevalent among rural indigenous community in Sarawak. Improvement of socioeconomic status, periodic mass deworming, iron supplementation and health education program should be included in the control and prevention of public health strategies.

## Introduction

Intestinal parasitic infections (IPIs) are among the most widespread health maladies in the developing world and on the World Health Organization (WHO) list of neglected tropical diseases (NTDs) [1]. IPIs are most prevalent among the poorest people [2–7], contributing to economic instability and social marginalization that can persist from generation to generation [1]. *Ascaris lumbricoides*, *Trichuris trichiura* and hookworms, collectively referred as soil-transmitted helminths (STHs) are the most prevalent of intestinal parasites. Approximately 24% of the world’s population is infected with at least one of species with an estimation of 135,000 deaths annually [8]. *Giardia lamblia*, causing giardiasis, is the most prevalent intestinal protozoan worldwide with an estimated prevalence rate ranging between 2 to 7% and 20 to 30% in developed and developing countries, respectively [9]. Another common intestinal protozoan is *Entamoeba histolytica*, causing amoebiasis which often leads to chronic intestinal infection and dissemination to the liver causing amoebic liver abscess (ALA) [2,5]. The opportunistic protozoan such as *Cryptosporidium* spp. are commonly reported among immunocompromised individuals with significant mortality and morbidity [2,5].

The relationship between IPIs particularly STHs, anaemia and malnutrition have been well documented [10–12]. They often share similar geographical areas. IPIs impair the nutritional status of those infected in many ways. These parasites can induce intestinal bleeding and competition for nutrients which leads to malabsorption of nutrients. They can also reduce food intake and ability to use protein and to absorb fat as well as increase nutrient wastage via vomiting, diarrhea and loss of appetite [11,12]. These effects lead to protein energy malnutrition, anemia and other nutrient deficiencies [11,12]. Such nutritional effects can have a significant impact on growth and physical development especially among school children and pregnant women as a result of heavy infections [10–16].

Although Malaysia has undergone rapid growth in socioeconomic and development infrastructure, IPIs and malnutrition are still endemic particularly among the underprivileged indigenous communities which are closely associated with their poor socioeconomic status (SES), personal and environmental hygiene [2,4,7,17–21]. Malaysia consists of Peninsular Malaysia and Borneo Malaysia. Most recent studies conducted among the indigenous communities in Peninsular Malaysia between 2013 and 2015 have reported overall prevalence of more than 50% [2,7,17,21]. To date, there are relatively limited data on the prevalence of IPIs among the indigenous groups in East Malaysia or Borneo Malaysia [22,23]. Moreover, almost all of these studies failed to take into consideration the possible associated risk factors among these indigenous communities. Within this context, the aim of the current study was to determine the current baseline prevalence and possible associated risk factors of IPIs, anaemia and malnutrition among the indigenous community in Sarawak. Findings of this study will fill vital gaps and provide beneficial insight and information on the epidemiology and disease dynamics of parasitic infections and its associated factors. Such data will be valuable for the public health authorities to justify and facilitate the reassessment of the existing control measures to reduce the prevalence and intensity of parasitic infections in these communities.

## Materials and Methods

### Study design and population characteristics

**Fig 1:**
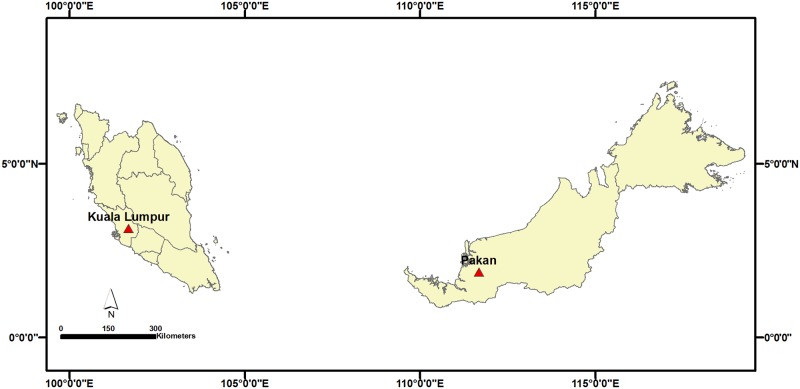
Location of the study area.

A cross-sectional study was carried out at three *Iban* tribe longhouses in the rural area of Pakan (1.88333°N, 111.68333°E) in Sarikei Division, Sarawak (Fig 1). The longhouses were selected based on (i) willingness to participate, (ii) road accessibility and (iii) village entry approval by the district officer. The sample size required for this study was calculated according to the anticipated prevalence of intestinal parasitic infections (IPIs) conducted among these communities [24]. According to the available community-based study conducted in Sarawak, the overall prevalence IPIs was 67.6% [22]. By using significance level of 5% and confidence level of 95%, a minimum sample size of 337 samples was required to achieve the objectives of this study.

A total of 341 participants comprising of 155 (45.5%) male and 186 (54.4%) female participated in this study. The age ranged from 1 year to 91 years with a median age of 30 years old and proportions of 43.4% and 56.6% for children (1–17 years) and adults (≥18 years), respectively. In general, the longhouses were located at a low altitude near jungle fringes surrounded by pepper plantation. Each longhouse comprises of 120 to 200 occupants. Each house within the longhouse is separated by a door. There can be up to 30 to 40 doors within a longhouse. A considerable number of school-going children resided in school hostels due to the far distance between their schools and the longhouse. The communities engaged mainly in agricultural work with no stable income. Up to 66.3% of the longhouse communities were categorized with low household income (<RM500/month) which is below the poverty line income. With regards to educational attainment, 34.6% participants mainly adults were with no formal education while the rest obtained either primary or/and secondary school. The provision of latrine facilities was available in 67.2% households. However, only 36.4% were pour flush toilets which were poorly maintained and mainly used by the adults while others defecated indiscriminately in bushes and river. Children generally defecated indiscriminately without parental supervision.

### Ethical approval, consent and questionnaire

The study protocol was approved by the Ethics Committee of the University Malaya Medical Centre (UMMC) (MEC ID: 201401–0672) and International Medical University (IMU) R162/2014 (MSc-UM), IMU Joint Committee on Research and Ethics. Prior to sample collection, parents and their children were given an oral briefing on the objectives and methodology of the study. The participants were informed that their participation was voluntary and could withdraw at any time without giving any reason. They were also informed that their identities and personal information will be kept strictly confidential. If they agree to participate, the informed consent form was signed for literate participant. For illiterate participant, their consent was obtained by their thumb print on the informed consent form. For children, the consent was obtained from their parents or guardians who are usually head of the family in similar approaches (i.e., signature or thumb print). Upon completion of consent form, the participants were subjected to answer a pre-designed questionnaire to collect information on their demography, socioeconomic status, behavioral aspects and basic medical treatment. The questionnaire was designed in the Malaysian national language, *Bahasa Malaysia* (S1 File). The questionnaire was completed by interviewing the heads of the household or any relevant adults.

### Fecal sample collection

Following the completion of consent and questionnaire, a wide screw capped containers pre-labeled with names were distributed to each respondent. The samples were collected on the following day. Participant who were not able to provide sample on the first day were asked again on the following day. The fecal samples were transported back to the laboratory in Hospital Sarikei for temporary storage. The samples were then couriered by air back to University of Malaya, Kuala Lumpur for parasitological analysis.

### Parasitological analysis

The fecal samples were subjected to formalin-ether sedimentation techniques [25]. In brief, 1 to 2g of sample was mixed with 7 ml of formalin and 3 ml of ether, centrifuged at 2500 rpm, and stained with iodine and examined under light microscope. The samples were also examined for *Cryptosporidium* spp., using modified Ziehl Neelsen staining technique which consists of 0.4% malachite green, 1% acid alcohol and strong carbol fuchsin. Approximately, 10g of feces fixed in polyvinyl alcohol (PVA) was thoroughly mixed and used to detect the presence of other intestinal protozoa using the Trichome staining technique. Stained samples were examined under light microscope at 100x magnification with oil immersion.

### Haemoglobin (Hb) measurement

Finger-prick blood samples were obtained from participant to assess Hb levels. Haemoglobin was measured in the field using a portable Hb analyzer (HemoCue^®^ Hb 201^+^, Angholm Sweden). The analyzer was calibrated against the international reference method for Hb concentration according to manufacturer’s instruction. The cut-off value for anaemia was determined according to the World Health Organization (WHO) guidelines [26]. Anaemia was confirmed when the Hb level is < 110 g/L for children aged 6 to 59 months, < 115 g/L for children 5 to 11 years, < 120 g/L for children 12 years [26].

### Anthropometric assessment

Height measurement was performed with the aid of a measuring tape. The respondents were requested to remove footwear, hair accessories that may obstruct measurement. A ruler was placed on top of their heads, and the reading was taken from the top of the ruler at eye level. Height was then read and recorded to the nearest 0.1 cm. A calibrated bathroom scale was used to measure weight. The device was placed on a level surface. Each respondent was asked to stand at the centre of the scale, barefoot with minimal clothing and empty pockets. The weight was recorded to the nearest 0.1 kg. To reduce intra individual error, height and weight were measured twice and the mean value was used for analysis.

Anthropometric indices were calculated using WHO Anthro software version 3.2.2 (WHO, Geneva, Switzerland). The anthropometric indices include: a) height for age (HAZ) to assess stunting; b) weight for age (WAZ) to access underweight c) body mass index for age (BAZ) to assess wasting. The values were expressed as differences from the median in standard deviation units (i.e., z-scores). Due to the inability to differentiate between relative height and body mass, WAZ is not recommended for the assessment of growth beyond childhood (˃10 years of age) as it could not distinguish between height and body mass in an age period of pubertal growth spurt and appearance of excess weight [27]. Therefore, BAZ was used as a complement to HAZ. Participant were classified as stunted, underweight and wasted if z-scores of HAZ, WAZ and WAZ were less than 2 standard deviations (S.D) below the National Center for Health Statistics reference (NCHS)/WHO median [27].

### Statistical analysis

Statistical analysis was carried out using the SPSS software (Statistical Package for the Social Sciences) program version 21 for windows (SPSS, Chicago, IL, USA). Data were reviewed and cross-checked before and after data entry by two different independent individuals for consistency. Descriptive data analysis was expressed as percentage and frequency. The distribution of quantitative variables was examined for normality using the Kolmogorov-Smirnov Z test before analysis. Mean and standard deviation (SD) were used to present the normally distributed variables whereas variables that were not normally distributed were expressed as median and interquartile range (IQR). A Chi Square (*X*^*2*^), One-way ANOVA, Pearson’s correlation coefficients (r_s_) and logistic regression analyses were used for statistical analyses. A *p* value less than 0.05 were taken to indicate statistical significance. In addition, a *p* value of 0.20 was used as an elimination criterion for regression analyses as suggested by Bendel and Afifi [28]. These authors demonstrated that use of more traditional level, such as *p*<0.05 for regression analyses often eliminated variables that later proved to be significant predicators.

## Results

### Prevalence and pattern of IPIs

The overall prevalence of IPIs was 57.5% (95% CI = 52.0–62.8%) with STH being significantly higher (50.4%; 95% CI = 45–55.9%) compared to protozoa (10%; 95% CI = 7.0–13.7%) infections. Of the STH species, *A*. *lumbricoides* (24.3%) was the most predominant, followed by hookworm (22%) and *T*. *trichiura* (14.4%) (Table 1). For protozoa, *Entamoeba* spp. was the most common (6.5%; 95% CI = 4.1–9.6%) followed by *G*. *lamblia* (3.5%) and *Cryptosporidium* spp. (0.3%) (Table 2). Of these, mono-parasitism was more common (40.2%) than poly-parasitism (10%). Poly-parasitism of *T*. *trichiura* and *A*. *lumbricoides* (4.1%) was the most frequent followed by *A*. *lumbricoides* and hookworm (3.8%) and *T*. *trichiura* and hookworm (1.8%). The prevalence of IPIs was further analyzed according to various age categories: young children (1 to 6years), primary school children (7 to 12years), teenagers (13 to 17 years) and adults (˃18 years). The overall prevalence of IPIs was generally varied from 33.3% to 100% in all age groups. The overall prevalence of STH (*p* = 0.037) and protozoan infections (*p* = 0.028) was significantly higher among young children.

**Table 1:** Prevalence of STH infections according to gender and age groups among indigenous longhouse communities.

		STH Infections											
Variables	N	*A*. *lumbricoides*				Hookworms				*T*. *trichiura*			
		n	%	95% CI	*p* value	n	%	95% CI	*p* value	n	%	95% CI	*p* value
**Gender**													
**Male**	155	37	23.9	17.4–31.4		34	21.9	15.7–29.3		24	15.5	10.2–22.2	
**Female**	186	46	24.7	18.7–31.6	0.854	41	22.0	16.3–28.7	0.981	25	13.4	9.0–19.2	0.592
**Age**													
**1 to 6**	39	8	20.5	9.3–36.5		8	20.5	9.3–36.5		4	10.3	2.9–24.2	
**7 to 12**	91	21	23.1	14.9–33.1		22	24.2	15.8–34.3		12	13.2	7.0–21.9	
**13 to 17**	18	3	16.7	3.6–41.4		0	0.0	0.0–18.5		2	11.1	1.4–34.7	
**> 18**	193	51	26.4	20.4–33.2	0.841	45	22.0	18.0–30.0	0.233	31	16.1	11.2–22.0	0.252
**Total**		83	24.3	20.2–30.0		75	22.0	17.7–26.8		49	14.4	10.8–18.6	

N: Total number examined; n: Number positive with STH infections

**Table 2:** Prevalence of protozoan infections according to gender and age groups among indigenous longhouse communities.

		Protozoan Infections											
Variables	N	*Entamoeba* spp.				*Giardia* spp.				*Cryptosporidium* spp.			
		n	%	95% CI	*p* value	n	%	95% CI	*p* value	n	%	95% CI	*p* value
**Gender**													
**Male**	155	7	4.5	1.8–9.1		3	1.9	0.4–5.6		0	0	0.0–2.4	
**Female**	186	15	8.1	4.6–13.0	0.184	9	4.8	2.2–9.0	0.147	1	0.5	0.0–3.0	0.361
**Age**													
**1 to 6**	39	4	10.3	2.9–24.2		2	5.1	0.6–17.3		0	0	0.0–9.0	
**7 to 12**	91	6	6.6	2.5–13.8		5	5.5	1.8–12.4		0	0	0.0–4.0	
**13 to 17**	18	4	22.2	6.4–47.6		0	0	0.0–18.5		1	5.6	0.1–27.3	
**> 18**	193	8	4.1	1.8–8.0	0.027*	5	2.6	0.9–6.0	0.183	0	0	0.0–1.9	0.001*
**Total**		22	6.5	4.1–9.6		12	3.5	1.8–6.1		1	0.3	0.0–1.6	

N: Total number examined; n: Number positive with protozoan infection

*Significant difference (*p*< 0.05)

### Prevalence of anaemia and malnutrition

The overall prevalence of anaemia was 36.4% (95% CI = 31.3–41.7%) with mean Hb of 126.0 g/L (SD ± 2.0). In general, anaemia was more common among the females (39.8%; 95% CI = 33–47%) than males (32.9%; 95% CI = 26–40.6%) (Table 3). The mean Hb in male was 121.1 g/L (SD ± 1.7) ranging from 67.0 g/L to 152.0 g/L. In female, the mean Hb was 129.0 g/L (SD ± 2.3) ranging from 60.0 g/L to 169.0 g/L. Further analysis demonstrated that the prevalence of moderate anaemia was higher in females (23.7%) compared to males (20%). Both mild and severe anaemia was also more frequent in female (13.4% and 2.7%) compared to male (11% and 1.9%), respectively.

**Table 3:** Prevalence of anaemia according to gender and age groups among indigenous longhouse communities.

		Anaemia#											
Variables	N	Mild				Moderate				Severe			
		n	%	95% CI	*p* value	n	%	95% CI	*p* value	n	%	95% CI	*p* value
**Gender**													
**Male**	155	17	11.0	6.5–17.0		31	20.0	14.1–27.1		3	1.9	0.4–5.6	
**Female**	186	25	13.4	8.9–19.2	0.489	44	23.7	17.8–30.4	0.417	5	2.7	0.9–6.2	0.647
**Age**													
**1 to 6**	39	3	7.7	1.6–20.9		11	28.2	15.0–44.9		3	7.7	1.6–20.9	
**7 to 12**	91	3	3.3	0.7–9.3		27	29.7	20.6–40.2		2	2.2	0.3–7.7	
**13 to 17**	18	3	16.7	3.6–41.4		2	11.1	1.4–34.7		2	11.1	1.4–34.7	
**> 18**	193	33	17.1	12.1–23.2	0.015*	35	18.1	13.0–24.3	0.017*	1	0.5	0.0–2.9	0.004*

N: Total number examined; n: number of anaemia;

*Significant difference (*p*< 0.05)

^#^ Classified according to the criteria proposed by WHO [22]

The prevalence of anaemia also showed an age dependency relationship with high prevalence reported among adults aged 18 years and above (55.2%), followed by school children aged 7 to 12years (25.6%) and young children aged 1 to 6 years (13.6%). The lowest prevalence was reported among teenagers aged 13 to 17 years (5.6%). It was reported that mild anaemia was significantly higher among adults aged 18 years old and above (17.1%; *p* = 0.015). Moderate anaemia was also significantly associated with school-going children aged 7 to 12 years (29.7%; *p* = 0.017). Meanwhile, severe anaemia was significantly higher among teenagers aged 13 to 17 years (11.1%; *p* = 0.004).

The overall prevalence of underweight (weight-for-age), wasting (BMI for age) and stunting (height-for-age) among children were 22.2% (95% CI = 16.2–29.7%), 5.6% (95% CI = 2.4–10.7%) and 35.4% (95% CI = 27.6–43.8%), respectively (Table 4). The mean for HAZ, BAZ and WAZ score was -1.53 (SD = 1.71), 0.21 (SD = 2.20) and WAZ score -0.74 (SD = 1.95). Underweight, wasting and stunting were generally more common among girls compared to boys. Only stunting showed an age dependency relationship with mean z-score of height and age was significantly higher among boys aged 1 to 6 years (*p*<0.001).

**Table 4:** Prevalence of anaemia and malnutrition (i.e., underweight, stunting and wasting) among indigenous longhouse children.

		Intestinal Parasitic Infections (IPIs)										
Variables	Mean (SD)	All children		Any IPIs			Mono-parasitism			Poly-parasitism		
		N = 144		N = 82			N = 58			N = 10		
		n	%	n	%	*p* value	n	%	*p* value	n	%	*p* value
**Nutritional Indicators**#												
**Mean HAZ**	-1.53 (1.71)	51	35.4	25	49.0	0.382	18	35.3	0.057	3	5.9	0.864
**Mean BAZ**	0.21 (2.20)	8	5.6	4	50.0	0.805	4	50.0	0.490	0	0	0.975
**Mean WAZ**	-0.74 (1.95)	32	22.2	14	43.8	0.982	13	40.6	0.510	3	9.4	0.676
**Mean Hb**	11.80 (1.85)	53	36.8	40	75.5	<0.001*	30	56.6	0.003*	4	7.5	0.515

N: Total number examined; n: Number of underweight, stunting and wasting

WAZ: weight-for-age (underweight), HAZ height-for-age (stunting), BAZ: weight-for-height (wasting)

^#^ Classified according to the criteria proposed by National Centre for Health Statistics reference (NCHS)

*Significant difference (*p*< 0.05); SD: Standard deviation for HAZ, BAZ, WAZ

### IPIs, anaemia and malnutrition

It is well documented that IPIs, anaemia and malnutrition are intertwined and co-exist especially among the rural and poor communities in developing countries. Against this background, this study also aims to determine the possible association of such variables among this indigenous community. In general, overall prevalence of STH was significantly associated with anaemia (*p*<0.001). Detailed analyses showed that prevalence of *A*. *lumbricoides* (*p* = 0.012), *T*. *trichiura* (*p* = 0.024) and hookworm (*p*<0.001) infections were found to be significantly associated with anaemia (Table 5). The results also demonstrated that STH infections was found to be significantly correlated with decreasing Hb value (r_s_ = 0.171, *p* = 0.002). Further analyses showed a significant correlation between *A*. *lumbricoides* (r_s_ = 0.112, *p* = 0.040), hookworm (r_s_ = 0.111, *p* = 0.041) and *T*.*trichiura* (r_s_ = 0.118, *p* = 0.029) with low Hb values.

**Table 5:** Prevalence of anaemia and STH infections among indigenous longhouse communities.

		STH Infections											
Variables	N	*A*. *lumbricoides*				Hookworm				*T*. *trichiura*			
		n	%	95% CI	*p* value	n	%	95% CI	*p* value	n	%	95% CI	*P* value
Anaemia	125	40	32.0	23.9–41.0		40	32.0	23.9–41.0		25	20.0	10.1–23.6	
Non-anaemia	216	43	19.9	14.8–25.9	0.012*	35	16.2	11.6–21.8	0.001*	24	11.1	7.3–16.1	0.024*

N: Total number examined; n: Number positive;

*Significant association (*p*< 0.05)

As for malnutrition, stunting was higher among children infected with STH (31.7%), followed by mono-parasitism (31%) and poly-parasitism (30%). However, this rate did not differ significantly (Table 6). Children who were underweight showed a similar trend, where infected children had a prevalence of 28.6%, those with monoparasitism was 28.3% and those with polyparasitism were 37.5%. A relatively low prevalence of wasting was observed among children infected with any type of STH (4.9%) compared to mono-parasitism (6.9%). However, no significant correlation was observed between stunting, underweight, wasting and infection status. In contrast, wasting was significantly correlated with mild anaemia (r_s_ = 0.188, *p* = 0.024).

**Table 6:** Nutritional indicators according to infection status among indigenous longhouse children.

	Intestinal Parasitic Infections (IPIs)										
Variables	All children		Any IPIs			Mono-parasitism			Poly-parasitism		
	N = 144		N = 82			N = 58			N = 10		
	n	%	n	%	*p* value	n	%	*p* value	n	%	*p* value
**Nutritional Indicators**											
**Stunted (<-2SD HAZ)**	51	35.4	26	31.7	0.284	18	31.0	0.367	3	30.0	0.710
**Wasting (<-2SD BAZ)**	8	5.6	4	4.9	0.683	4	6.9	0.564	0	0	0.427
**Underweight (<-2SD WAZ)**	32	31.7	18	28.6	0.387	13	28.3	0.499	3	37.5	0.712

N: Number examined; n: Number positive

The association of IPIs and socioeconomic status (SES) was examined by univariate analysis as shown in Table 7. The results showed that absence of toilets (OR = 2.0; 95% CI = 1.3–3.3%; *p* = 0.003), close contact with animals (OR = 2.6; 95% CI = 0.8–9.0%; *p* = 0.030), taking bath once a day (OR = 1.2; 95% CI = 1.1–1.6%; *p* = 0.028) and changing clothes once a day (OR = 1.3; 95% CI = 1.1–1.6%; *p* = 0.017) were significantly associated with IPIs. The final multivariate analysis indicated that the absence of toilets (OR = 1.6; 95% CI = 1.1–2.7%; *p* = 0.002) and close contact with animals (OR = 1.8; 95% CI = 1.3–2.9%; *p* = 0.027) as significant predictors for IPIs.

**Table 7:** Potential risk factors associated with intestinal parasitic infections (IPIs) among indigenous longhouse communities (Univariate analysis, N = 341).

		IPIs			
Variables	N	n	(%)	OR (95% CI)	*p* value
**Age in years**					
≥12	132	79	59.8	1.1(0.7,1.1)	
<12	209	117	56.0	1	0.482
**Gender**					
Male	155	92	59.4	1.2 (0.7,1.8)	
Female	186	104	55.9	1	0.522
**Education levels**					
Non educated	118	71	60.2	1.2(0.8, 1.9)	
Educated	223	125	56.1	1	0.465
**Employment status**					
Unemployed	210	119	56.7	0.9(0.6,1.4)	
Employed	131	77	58.8	1	0.701
**Household income**					
< RM500	226	127	56.2	0.9(0.5,1.4)	
≥ RM500	115	69	60.0	1	0.502
**Presence of toilet in house**					
No	112	77	68.8	2.0(1.3,3.3)	
Yes	229	119	52.0	1	0.003*
**Indiscriminate defecation**					
Yes	217	112	56.2	0.9(0.6,1.4)	
No	124	74	59.7	1	0.535
**Presence of domestic animal**					
Yes	328	190	57.9	1.6(0.5,4.9)	
No	13	6	46.2	1	0.400
**Eating with hands**					
Yes	338	195	57.7	1.7(0.3,8.6)	
No	3	1	33.3	1	0.396
**Close contact with domestic animals**					
Yes	332	194	58.4	2.6(0.8,9.0)	
No	9	2	22.2	1	0.030*
**Iron supplement**					
No	331	188	56.8	0.7(0.5,1.0)	
Yes	10	8	80.0	1	0.144
**Anthelminthic drugs**					
No	291	164	56.4	0.7(0.4,1.4)	
Yes	50	32	64.0	1	0.313
**Taking bath once a day**					
No	63	44	69.8	1.2(1.1,1.6)	
Yes	278	152	54.7	1	0.028
**Changing clothes once a day**					
No	54	39	72.2	1.3(1.1,1.6)	
Yes	287	157	54.7	1	0.017
**Wearing shoes outside the house**					
No	160	88	55.0	0.9(0.5,1.3)	
Yes	181	108	59.7	1	0.384
**Washing hands after defecation**					
No	56	30	53.6	0.9(0.5,1.5)	
Yes	285	166	58.2	1	0.518
**Washing handsbefore and after playing with soil**					
No	66	35	53.0	0.8(0.5,1.4)	
Yes	275	161	58.5	1	0.416

N: Total number examined; n: Number of IPIs

Reference group marked as OR = 1; CI: Confidence interval

Significant association (*p*< 0.05)

*Variables were confirmed by multivariate analysis as significant predictors of IPIs

No formal education (OR = 1.5; 95% CI = 1.1–2.0%; *p* = 0.006) was significantly associated with anaemia as examined by univariate analysis (Table 8). Detailed analysis of the infection status showed that poly-parasitism of *T*. *trichiura* and hookworm were significantly associated with anaemia (OR = 1.1; 95% CI = 1.3–9.0%; *p* = 0.017). As for malnutrition, low household income (<RM500) was identified as a significant predicator for stunting (OR = 2.1; 95% CI = 9.8–22.2%; *p*<0.001) and underweight (OR = 1.9; 95% CI = 5.6–18.7; *p* = 0.037), respectively.

**Table 8:** Potential risk factors associated with anaemia among indigenous longhouse communities (Univariate analysis, N = 341).

		Anaemia			
Variables	N	n	(%)	OR (95% CI)	*p* value
**Age in years**					
≥12	132	48	36.4	0.9(0.6,1.5)	
<12	209	77	36.8	1	0.929
**Gender**					
Female	186	74	39.8	1.1(0.9,1.3)	
Male	155	51	32.9	1	0.189
**Education levels**					
Non educated	118	55	46.6	1.5(1.1,2.0)	
Educated	223	70	31.4	1	0.006*
**Employment status**					
Unemployed	210	84	40.0	1.2(1.0,1.7)	
Employed	131	41	31.3	1	0.105
**Household income**					
< RM500	226	81	35.8	0.9(0.7,1.3)	
≥ RM500	115	44	38.3	1	0.661
**Iron supplement**					
No	331	123	37.2	1.9(0.5,6.5)	
Yes	10	2	20.0	1	0.267
**Anthelminthic drugs**					
No	291	107	36.8	1.1(0.7,1.6)	
Yes	50	18	36.0	1	0.917

N: Number examined; n: Number positive

Reference group marked as OR = 1; CI: Confidence interval

Significant association (*p*< 0.05)

*Variables were confirmed by multivariate analysis as significant predictors of anaemia

## Discussion

Intestinal parasitic infections (IPIs) remain a major public health problem among the Malaysian indigenous population where poor environmental and sanitation, improper hygiene, overcrowding, low education attainment and poverty are common. The findings of the present study showed a high prevalence of IPIs with 57.5% of participants was infected with at least one species. These findings in a state in Borneo Malaysia were relatively consistent to the report from previous study among indigenous groups in Peninsular Malaysia with prevalence rates of 50% and above [2,4,7,19–21]. Similarly, the results were also consistent with previous local study conducted among other indigenous community in Saratok Division which reported high prevalence of IPIs (67.6%) [28]. However, these findings were contrary to earlier studies conducted among various indigenous groups in Kapit and Serian Division with low prevalent rate (below 50%) [22,23]. This variation may be due to differences in the sampling population, sample size, socioeconomic status(SES) of the participants and environmental sanitation.

Finding of this study demonstrated that prevalence of IPIs was significantly higher among children compared to adults. Much epidemiological research suggests that IPIs exhibit distinct age-dependent relationship patterns [29,30]. The changes with age in the frequency and intensity of infections generally tend to be convex, rising in childhood and declining in adulthood [31]. For *A*. *lumbricoides* and *T*. *trichiura*, the prevalence and intensity rises rapidly and peaks during childhood before declining gradually in adulthood [32]. In contrast, hookworm exhibits a steady rise in the frequency and intensity of infections with age, commonly peaks in adulthood or even in elderly people [33]. Whether such age dependency is due to exposure, acquired immunity or a combination of both remains unresolved [34].

The possible explanation could be due to the fact that as the child grows older, the exposure to the sources of infection increases. As for children, they are more independent, active and inquisitive and are interested in learning new things in their surroundings. Due to their young age, many are still not fully aware of personal hygiene and good cleanliness practices or to realize the consequence of exposing themselves to pathogenic organisms. However, this linear association of age-dependent relationship patterns still needs further exploration through prospective studies.

Some of the strongest evidence for protective immunity in the helminthic infections in human has come from epidemiological observations in the frequency and intensity of infections with age, called the ‘peak shift’ [35]. Briefly, this theory suggested that if age-infection data are compared across host populations, the peak level of infection frequency and intensity is higher and occurs in younger individuals when the transmission rate is high. In contrast, the peak level of infection frequency and intensity is lower and occurs in older individuals when transmission is low [35]. The ‘peak shift’ theory is consistent with the predictions of mathematical models that assume gradually acquired protective immunity. This finding is further supported by experimental studies in animals [35].

The effect of poor socioeconomic status on parasitic infection in particular, is complex in nature and closely associated with poor environmental sanitation, personal and sanitary behavior, overcrowding and impoverished health services [36,37]. As a result, IPIs are generally co-endemic and may differ from one region to another and sometimes within the country itself because of the variation between the associated risk factors [38]. This study has showed that the SES of the indigenous community remains generally poor and appeared to be similar across all longhouses. Previous local studies have shown that such webs of risk factors were significantly associated with high prevalence of IPIs among rural and indigenous communities [2,4,7,19–23], findings that were in agreement with the present study.

In the present study, a house-hold toilet facility was almost absent. Single pit latrines which are dug few meters into the ground near the longhouse often shared between few households. However, it was rarely used because of poor maintenance, which encourages indiscriminate defecation. Villagers were observed defecating indiscriminately close to their houses or within the village confines including in the river. This may indirectly contaminate the surrounding areas. In addition, few studies assessing the impact of pit latrines on ground water quality demonstrated that pit latrine as a direct source of water contamination of the subterraneous water flow that passes through the fecal-infected soil into the river [37, 39]. As the longhouses are located close to a river where the occupants have easy access to water supply for daily chores, this may increase the risk of transmission and infection particularly waterborne protozoan parasites [40]. Thus, it was not surprising that absence of toilet was a significant predictor for IPIs among this community. The present study also demonstrated close contact with animals was significantly associated with IPIs, a finding that is consistent with previous studies [41–43]. Although the notion of zoonoses of IPIs is remain largely unclear, animal may acts as mechanical transmitter in the human population especially in communities where the habit of indiscriminate defecation exits [41–43].

Comparing the findings of the present study with other local studies demonstrated that the prevalence of anaemia (36.7%) was higher than that reported previously in Sarawak (24.4%) [44] and another Borneo Malaysia state (Sabah; 20%) [45]. In contrast, higher rates were observed in other local studies conducted in Peninsular Malaysia with prevalence ranging from 41.5% to 48.5% [18,19]. Globally, low prevalence of anaemia were reported among rural community in Brazil (11.8%) [46] and South Africa (16.5%) [47]. WHO has proposed the following classification of populations with respect to the level of public health significance of anaemia: a prevalence of <19.9% is considered to be ‘low’, 20% to 39.9% is ‘moderate’ and ≥40% as severe [26]. Therefore, findings of this study reflected that anaemia is still a public health problem among the indigenous communities in the longhouses in Sarawak.

It is well documented that IPIs particularly STH infections were significant predictors for anaemia and malnutrition, especially in children [10–13, 18–20]. The present study showed that STH infections were significantly associated with anaemia, a finding that is in agreement with other local studies conducted among rural children [18–20]. The present study showed that co-infection of *T*. *trichiura* and hookworm was significantly associated with anaemia which is in agreement with a study conducted in Panamanian children [12]. This could be due to the synergistic effect of both species causing blood loss by hookworm and impaired re-absorption or ingestion of iron by *T*. *trichiuria*.

This study highlighted that anaemia was more common among females than males. These findings were consistent with previous local studies [18,19,44,45]. Generally, females are more likely to be anaemic compared to male especially during the reproductive age due to their physiological changes and due to menstrual blood loss. This study also indicated that anaemia was significantly associated with age groups. This finding is in agreement with a study conducted among rural children in Malaysia where anaemia was higher among children [19]. A survey conducted by the Ministry of Health Malaysia reported 20% of children age less than 5 years old had low haemoglobin concentrations which indicated moderate anaemia [48].

In Malaysia, the impact of IPIs on nutrition, growth and development of indigenous communities has been studied extensively since the late 1980s [49]. Malnutrition is often associated with anaemia due to low intake of heme iron from animal food sources as well as a result of poor diet quality and poverty. These conditions are further aggravated with STH infections, malaria, and other blood-destroying infection [10–12]. WHO estimated that Asia region has the highest number of malnourished children in the world [50]. Stunting (i.e., indicator of past or chronic malnutrition) was found to be prevalent among the indigenous school children followed by underweight and wasting. Although WHO has estimated that an overall prevalence of stunting has decreased in developing country [50], this study highlighted that stunting still remains as a major public health problem among this indigenous children.

This result was also consistent with earlier findings in Malaysia showing high prevalence of stunting and underweight among the indigenous children [48,49,51]. Stunting showed an age dependency relationship among children especially ages between1 to 6 years old, with inadequate feeding or the presence of recurrent illness or chronic illness [51]. Children in rural communities are at greater risk of becoming stunted than children living in urban areas. Previous studies conducted among the Malaysian primary school children indicated that children in rural areas had significantly higher prevalence of malnutrition than urban areas [49,51] and similar to the results obtained in this study.

The present study also found that wasting was significantly associated to mild anaemia. It was reported that mild to moderate anaemia was always present in cases of severe malnutrition [51]. On the other hand, this study showed that socioeconomic factor such as low household income was significantly associated with stunting and underweight. This was in agreement with the previous local study that reported a significant association between low household income and malnutrition [51]. School-going children have access of school-based health care, including periodic health care monitored by the health care team and supplementary feeding programs. However, much younger children have limited access to health care especially in disadvantage communities thus this correlated to our findings which showed stunting were more prevalent among children aged 1 to 6 years. In the present study, however the type of food consumed and the daily energy and protein intake were not documented nor measured. A study conducted among the children of indigenous communities revealed that daily energy and the protein intake were below RDI (Recommended Daily Intake) and were significantly associated with malnutrition [51].

One of the main limitations faced in this study was that only a single stool sample was examined instead of three samples consecutively due to lack of response among the longhouse communities. Hence, there is a possibility of underestimation of the true prevalence rate of IPIs. A second possible limitation was the interviewer bias during administration of the questionnaire. Nonetheless, attempts to minimize these limitations were emphasized by standardizing interviewer’s interaction with respondents by training the interviewers. In addition, the same interviewers were appointed throughout the study. Thirdly, this study only used formalin ether concentration technique with the absence of egg count data. Previous study indicated that dietary iron insufficiency has significant impact on Hb and anaemia [52]. However, information on the iron status of the children was not assessed in the present study, a subject that needs to be incorporated in future study.

## Conclusions

This study provided baseline data on the intestinal parasitic infections (IPIs), anaemia and malnutrition among indigenous community in rural areas in Sarawak, Borneo Malaysia. Generally, the IPIs, anaemia and malnutrition are still public health problems among this indigenous community. IPIs were found to be significantly associated with anaemia. Meanwhile, the incidence of malnutrition (i.e., stunting, underweight and wasting) was higher among infected children with IPIs. Absence of toilets and close contact with animals was identified as significant predictors for IPIs. No parental formal education was significantly associated with anaemia. As for malnutrition, low household income was identified as a significant predicator for both stunting and underweight. This data strongly support the need for implementing health program aimed at improving nutrition, anaemic conditions and reducing the prevalence of infections, with potentially beneficial effects on cognitive and educational outcomes particularly among children. With effective control measures in place, these children will have a greater opportunity for a better future in terms of health and educational attainment, which will eventually put them on par socially and economically with other communities in Malaysia.

## Supporting Information

S1 File Questionnaire (English-translated version). (DOCX)
