# Supplementary material for: Neglected Intestinal Parasites, Malnutrition and Associated Key Factors: A Population Based Cross-Sectional Study among Indigenous Communities in Sarawak, Malaysia
Source: PLoS One. 2017 Jan 17;12(1):e0170174. doi: 10.1371/journal.pone.0170174 (PMC5240947; doi:10.1371/journal.pone.0170174)
Supplement: S1 File — (DOCX) [file pone.0170174.s001.docx]

**QUESTIONNAIRE FORM**

**Sampling Date:**

**Village Name:**

**Unit/House No:**

**A. Socio-Demographic**

| No | Name | DOB  (d/m/y) | Age | Gender | Educational attainment | Occupation | Household  Income | Height  (cm) | Weight  (kg) |
| --- | --- | --- | --- | --- | --- | --- | --- | --- | --- |
| 1. |  |  |  |  |  |  |  |  |  |
| 2. |  |  |  |  |  |  |  |  |  |
| 3. |  |  |  |  |  |  |  |  |  |
| 4. |  |  |  |  |  |  |  |  |  |
| 5. |  |  |  |  |  |  |  |  |  |
| 6. |  |  |  |  |  |  |  |  |  |

**Age**

1 = < 1 year

2 = 1- 4 years (Toddler)

3 = 5 - 6 years (Preschool)

4 = 7 - 12 years (Primary school)

5 = 13 – 17 years (Secondary school)

6 = > 18 years (Adults)

**Gender**

M = Male F = Female

**Educational attainment**

1 = No formal education

2 = Primary school

3 = Secondary school

4 = Other (please specify :___________________________)

**Occupation**

1 = Jungle product gatherer

2 = Rubber tapper

3 = Government employee

4 = Factory

5 = Palm oil plantation

6 = Labor (construction worker)

7 = Small business (mini sundry shop)

8 = Housewife/Not working

9 = Other (please specify :___________________________)

**Household Income (RM/month)**

1 = < RM 500

2 = >RM 500

**B. Environmental and Sanitation**

**Source of water supply**

1 = Government pipe water

2 = River

3 = Well

4 = Rain water

* More than one answer is allowed

**Sanitation**

**Presence of latrine in the house**

1 = Yes

2 = No

**Type of toilet facility**

1 = Pour flush toilet available

2 = Pour flush toilet not available

**Defecation site**

1 = Pour flush toilet

2 = Pit latrine

3 = River

4 = Bush

5 = Other (Please specify :_________________________)

* More than one answer is allowed

**Garbage disposal**

1 = Collected

2 = Indiscriminately

**Presence of domestic animal**

1 = Yes

2 = No

**Type of animal**

1= Dogs

2= Cats

3= Chicken

4= Duck

5= Others (Please specify :_________________________)

* More than one answer is allowed

**Close contact with domestic animal**

1 = Yes

2 = No

**C. Personal Hygiene**

**Option answers for question (a) to (f):**

1 = Yes

2 = No

1. Do you eat with hands?
2. Do you bath at least once a day?

1. Do you change your clothes at least once a day?
2. Do you wear shoes when you go outside?
3. Do you wash your hand before eating soon after playing with soil?
4. Do you wash your hand after defecation?
